# Bacteria associated with glioma: a next wave in cancer treatment

**DOI:** 10.3389/fcimb.2023.1164654

**Published:** 2023-05-02

**Authors:** Yiming Meng, Jing Sun, Guirong Zhang, Tao Yu, Haozhe Piao

**Affiliations:** ^1^ Department of Central Laboratory, Cancer Hospital of Dalian University of Technology, Liaoning Cancer Hospital & Institute. No. 44, Shenyang, China; ^2^ Department of Biobank, Cancer Hospital of Dalian University of Technology, Liaoning Cancer Hospital & Institute. No. 44, Shenyang, China; ^3^ Department of Medical Imaging, Cancer Hospital of Dalian University of Technology, Liaoning Cancer Hospital & Institute. No. 44, Shenyang, China; ^4^ Department of Neurosurgery, Cancer Hospital of Dalian University of Technology, Liaoning Cancer Hospital & Institute. No. 44, Shenyang, China

**Keywords:** bacteria, glioma, treatment, nanoparticle, drug delivery systems

## Abstract

Malignant gliomas occur more often in adults and may affect any part of the central nervous system (CNS). Although their results could be better, surgical excision, postoperative radiation and chemotherapy, and electric field therapy are today’s mainstays of glioma care. However, bacteria can also exert anti-tumor effects *via* mechanisms such as immune regulation and bacterial toxins to promote apoptosis, inhibit angiogenesis, and rely on their natural characteristics to target the tumor microenvironment of hypoxia, low pH, high permeability, and immunosuppression. Tumor-targeted bacteria expressing anticancer medications will go to the cancer site, colonize the tumor, and then produce the therapeutic chemicals that kill the cancer cells. Targeting bacteria in cancer treatment has promising prospects. Rapid advances have been made in the study of bacterial treatment of tumors, including using bacterial outer membrane vesicles to load chemotherapy drugs or combine with nanomaterials to fight tumors, as well as the emergence of bacteria combined with chemotherapy, radiotherapy, and photothermal/photodynamic therapy. In this study, we look back at the previous years of research on bacteria-mediated glioma treatment and move forward to where we think it is headed.

## Introduction

Approximately 80% of adults’ malignant tumors of the CNS are gliomas. Fifty percent of gliomas are grade 4 glioblastomas (GBM). Patients with GBM have a median survival time of 14 months, even after complete surgical resection and other treatments like chemotherapy and radiation(McKinnon et al., 2021; Miska and Chandel, 2023). Moreover, since radiation and chemotherapy are ineffective due to the milieu of hypoxia, high osmotic pressure, and immunosuppression, there is an urgent need to create novel anti-glioma therapeutic strategies.

The effective utilization of microorganisms to cure tumors provides optimism for treating tumors. Since the 19th century, when William B. Coley successfully treated patients with inoperable sarcomas with Streptococcus pyogenes, bacteria have been explored as anticancer agents (Evdokimova et al., 2022). Several bacterial species have had their utility in treating cancer enhanced by genetic engineering thanks to advances in synthetic biological methods. Compared to conventional cancer treatments, the characteristics of bacteria genetically created for this purpose are particularly noteworthy. They can localize accumulation inside tumors and halt cancer development (Li et al., 2023; Shen et al., 2023). Radiation and chemotherapy may be more successful when paired with genetically altered bacteria to function as a vector for the delivery of antitumor drugs (Mayakrishnan et al., 2022). However, the FDA has only authorized bacillus Calmette-Guérin (BCG), an attenuated strain of Mycobacterium bovis, as an anticancer bacterial agent to treat non-invasive bladder cancer (Kowald et al., 2023).

## Potential future applications for bacterial therapies

An intricate milieu is created for tumor development by combining hypoxia, a low pH, high permeability, and plentiful nutrition (Li et al., 2020). Salmonella, Escherichia coli, Bifidobacterium, and Streptococcus pyogenes are just a few of the obligatory and facultative anaerobes that may utilize their chemoreceptors to detect low levels of oxygen. They are naturally targeted to tumors in hypoxic and necrotic regions. In addition to targeting molecular targets such as tumor proteins and antigens, bacteria-based treatment may also target tissue-level pathological alterations (Alam et al., 2022). Employ recombinant DNA technology to boost the antitumor impact of engineered bacteria *via* directed genetic alteration (Xia et al., 2020). Studies have shown that engineering Salmonella with the lymphoma-associated antigen CD20 may effectively prevent malignancies and considerably decrease endogenous accumulation in the spleen and liver (Abedi et al., 2022). Escherichia coli may be formed into appropriate modular synthetic cohesion model bacteria for a range of tumor-specific ligands to produce anti-tumor effects (Robledo et al., 2022).

Due to inadequate lymphatic drainage and blood vascular penetration, tumor tissue often displays a high interstitial fluid pressure. It is difficult for many chemotherapy medications to overcome resistance and enter deeply into the tumor (Maleki Dana et al., 2022). In addition, the circulatory system at the tumor location is disordered, and the distance between capillaries is excellent; both hamper medication delivery (Kashkooli et al., 2023). Due to their status as sentient beings, bacteria can move about independently and actively migrate through tumor tissues(Salem-Bekhit et al., 2021). It could be an effective drug carrier that more than makes up for the inefficiencies of standard chemotherapeutic drug delivery. Tumor-associated bacteria may trigger host cell cytokine production, create inflammatory cascades, and prime the body for anti-tumor immune responses (Deng et al., 2018; Arai et al., 2022). Salmonella can induce the body’s innate immune response, activate dendritic cells, neutrophils, macrophages, and other immune cells to migrate to the tumor area, and enhance inflammatory cytokines resulting in an anti-tumor immune response (Bearson, 2022). Tumor-targeted engineered bacteria may boost anti-tumor immunity by changing how immune proteins are distributed in the body and turning on the Toll-like receptor signaling cascade. The optimized genetic circuit parameters in a probiotic bacterial system by Gurbatri et al. allowed for the precise and potent release of cytotoxic T lymphocyte-associated protein-4 (CTLA-4) and programmed cell death-ligand 1 (PD-L1), resulting in a significant increase in the therapeutic efficacy for tumor regression (Gurbatri et al., 2020). Bacterial therapy is superior to standard tumor treatment in specificity, manageability, and oncolytic efficacy (Kim et al., 2023b). It effectively reverses cancer and increases the body’s chance of living longer because it can target tumors and change genes, making a wide range of anti-tumor proteins. This method complements standard cancer treatment and is thus excellent for targeted tumor therapy.

## Preliminary study on the link between glioma and bacteria

Microorganisms are more numerous than human cells within and outside the body. There seems to be a far more vital link between some bacteria and cancer than was previously believed(Pandey et al., 2023). The brain regulates the composition and behavior of gut microbiota on the one hand. On the other hand, the intestinal flora regulates the brain through several pathways, including the endocrine, immunological, neuroendocrine, and metabolic systems, constituting the “gut flora-gastrointestinal-brain axis.” (Lyu et al., 2022; Pandey et al., 2023) Significant changes were demonstrated between mice and glioma patients regarding beta diversity, Firmicutes/Bacteroides (F/B) ratio, and expansion of the Verrucomicrobia phylum and Akkermansia genus. In a mouse model, glioma growth revoked dysbiosis of the intestines(Patrizz et al., 2020). In addition, Giuseppina D’Alessandro et al. reported the changes in the intestinal flora and immune changes following the treatment of glioma mice with two antibiotics(D'Alessandro et al., 2020): a reduction in the type and number of intestinal microbiota at the family level, a decline in the population of cytotoxic NK cell subsets; and an alteration in the expression of inflammatory and homeostatic proteins in microglia. It was shown that the formation of intracranial gliomas in mice treated with two antibiotics was considerably accelerated compared to animals not treated with antibiotics. These findings imply that long-term treatment with antibiotics modifies the microbiota makeup and helps manage the brain’s immunological state, facilitating the establishment of gliomas. The incidence further proves a strong association between gut microorganisms and glioma.

Researchers have also discovered a correlation between the gene function of the oral microbial population and glioma tumor grade(Wen et al., 2021). Oral bacteria are an integral element of the human microbiome and have been linked to various oral and systemic disorders (Chen et al., 2023a). Consistent with the findings of glioma malignancy, research has shown a clear correlation between the glioma IDH-mutant and the genera Bergeyella and Capnocytophaga. The IDH1-mutant is crucial for the induction of glucose, the metabolism of glutamine, the creation of lipids, and the control of cellular redox homeostasis in glioma cells (Carney et al., 2023). Their study showed that IDH-mutant people had a significantly higher enrichment of oral microbial gene functions related to lipid metabolism and the AMPK signaling pathway. The quantity of the oral phylum Patescibacteria was drastically reduced during glioma malignancy. Furthermore, their findings highlight the potential of the Patescibacteria phylum as a diagnostic and prognostic tool for GBM; however, more trials are required to examine the mechanisms behind these associations. In addition, the abundance of the Fusobacteria phylum in the group with high-grade glioma was considerably lower than that in the healthy control group. Leptotrichiaceae and the genus Leptotrichia were also adversely linked with glioma malignancy.

## Glioma therapy and the microbiome

Microorganisms, such as bacteria and fungi, are part of the microbiome, which includes the microbial community of the human digestive tract, skin, and other tissues (Berg et al., 2020). Innovations in illness therapy are emerging due to groundbreaking studies of the microbiome. One of the first microbiome treatments was feces-derived microbiota transplantation (FMT) (Lee et al., 2022). Small chemicals, biological agents, phages, and modified bacteria are only some of the novel agents produced by scientists to control the human microbiome and halt or alter the development of illness. Microbiome modification has expanded from its original use in treating gastrointestinal problems to many other diseases, including cancer and neurological disorders like Alzheimer’s and Parkinson’s (Abedi et al., 2022; Korczak et al., 2023). ENTEROME is a biopharmaceutical firm in the clinical development stage that uses insights into the microbiome-immune-inflammation axis to create new treatments for gastrointestinal diseases. The first human study of the cancer immunotherapy EO2401 based on microbiome antigen (OncoMimic) has begun. The effectiveness and safety of EO2401 in combination with immune checkpoint inhibitors for treating recurrent GBM will be studied in phase I/II clinical study. In an orthotopic mouse glioma model, Bifidobacterium (B.) lactis and Lactobacillus (L.) plantarum decreased tumor volume, lengthened survival time, and restored intestinal barrier damage, according to further study. Experiments revealed that the combination of B. lactis and L. plantarum inhibited the PI3K/AKT pathway and decreased the production of Ki-67 and N-cadherin (Wang et al., 2022). There has been a rapid development of microbiome-based medications. On the one hand, microbial therapies may supply physiological quantities of chemicals to the host and are anticipated to offer a long-term therapy alternative. Additionally, live bacteria can turn on different signaling pathways in the host, which could make therapies work better in the future(Gilbert et al., 2018; Yuan et al., 2023). Treatments based on the microbiome are now being researched for infectious diseases, metabolic diseases, central nervous system disorders, cancer, and other diseases. Therefore, the microbiome treatment will introduce us to a new universe (Krohn et al., 2022; Kunath et al., 2022).

## Bacterial components in glioma treatment

Cytotoxic necrotizing factor 1 (CNF1), a protein toxin from Escherichia coli, is surprisingly efficient as an anti-neoplastic treatment in a mouse model of glioma, decreasing tumor volume while improving survival and preserving the functional features of peritumoral neurons (Vannini et al., 2014; Vannini et al., 2016; Maroccia et al., 2018; Tantillo et al., 2018). However, due to its inability to traverse the blood-brain barrier (BBB), CNF1 must be injected directly into the brain, a very invasive administration method. Andrea Colarusso et al. (Colarusso et al., 2020) have taken advantage of a CNF1 mutant, including an N-terminal BBB-crossing tag, which was created to circumvent this hazard. They made the variation and determined whether its activity in GBM cells was equal to that of wild-type CNF1. They studied the signaling pathways in the cell’s response to CNF1 variations to give preliminary data for future animal investigations. CNF1 may be a unique therapeutic option for GBM because it protects the structure and function of healthy surrounding tissue and inhibits tumor development. This makes CNF1 the most intriguing choice among potentially useful bacterial toxins for treating brain malignancies. To enable the non-invasive delivery of CNF1, Eleonora Vannini et al. (Vannini et al., 2021) created a chimeric protein (CTX-CNF1) by conjugating CNF1 with chlorotoxin (CTX), a peptide currently used in clinical settings owing to its capacity to cross the BBB and attach specifically to glioma cells. After systemic injection, they discovered that CTX-CNF1 could target glioma cells and considerably extend the longevity of mice with glioma. The results of their study raise hope that CTX-CNF1 may one day serve as an innovative and highly efficient therapy for gliomas.

## Bacteria-based drug delivery systems

Creating an effective DDS that can penetrate the BBB, ordinarily impervious to most medications, is a significant obstacle in treating GBM (Cui et al., 2023). There have been proposals for both invasive and non-invasive medication delivery methods to the brain (Wang et al., 2023c). Deep brain stimulation, interbrain transplantation, direct brain injection, intrathecal brain delivery, and other techniques fall under the “invasive.” Non-invasive methods, such as receptor-mediated transcytosis, neurotropic viruses, exosomes, nanoparticles, etc., have gained popularity as an alternative to the invasive procedures that carry such a high risk of infection and suffering for the patient (Alberto et al., 2022; Lofts et al., 2022). In this regard, nanoparticle drug delivery devices are a benign option with therapeutic potential in treating GBM. Its appealing characteristics include high drug loading efficiency, geographically and temporally regulated drug release, real-time visualization during therapy, etc. (Tanaka et al., 2023). However, there are no clinically approved nanoparticle-based therapies for GBM: (1) nanoparticles’ size, surface charge, and opsonization may influence phagocyte absorption, inhibiting their entry into the brain. (2) The therapeutic drug is often loaded onto nanoparticles by charge interaction or hydrophobic contact, which may result in premature payload release during systemic circulation. (3) Due to the high interstitial fluid pressure of GBM tissue, even nanoparticles that pass through the BBB have difficulty penetrating deeply into GBM tissue, limiting their therapeutic benefits (Ahmad et al., 2023; Kim et al., 2023a; Kumar et al., 2023). In contrast, bacteria have lately shown promising successes in cancer treatment. However, several obstacles still exist, including the inability to accurately regulate medication delivery, inadequate activation of the immune system, and the possibility of bacterial damage (Chen et al., 2022a). Consequently, there is a rising interest in constructing hybrid bacteria-nanoparticle systems for medication delivery against different forms of cancer, which is rather intriguing (Dong et al., 2018). However, in these systems, the nanoparticles are often placed on the bacterium’s surface. Thus, they are still susceptible to the drawbacks mentioned above associated with nanoparticles that are free to move. Moreover, surface-loaded nanoparticles may compromise the integrity of bacterial capsules (Jimenez-Jimenez et al., 2022). The intact pills impede the fusion of bacteria with lysosomes, which is required for bacteria to cross the BBB as live organisms. A bacteria-nanoparticle hybrid approach against GBM across the BBB has yet to be developed. Sun et al. (Sun et al., 2022) designed a “Trojan” bacterial system to get therapeutic molecules into the brain for photothermal immunotherapy of GBM. The therapeutic drug comprises indocyanine green (ICG)-loaded glucose polymer (GP)-conjugated silicon nanoparticles (GP-ICG-SiNPs). Through bacteria-specific ATP-binding cassette (ABC) transporters, GP-ICG-SiNPs may be selectively and robustly ingested by facultative anaerobic bacteria such as Escherichia coli 25922 (EC), resulting in a Trojan bacterial system. The modified Trojan bacteria can transport the treatment over the BBB, target the GBM, and then penetrate the GBM tissue more deeply than free therapy, which has trouble entering the brain and piercing the GBM tissue. ICG molecules placed on SiNPs may transform light energy into sufficient heat to kill tumor cells and stimulate the release of tumor-associated antigen (TAA) when exposed to 808 nm light. Simultaneously, the produced heat may lyse host bacterial cells and facilitate the release of several pathogen-associated molecular patterns (PAMPs). PAMPs may stimulate the activation of macrophages and natural killer (NK) cells, resulting in innate anti-tumor immunity. On the other hand, the generated PAMPs may increase tumor infiltration frequency by activating CD8^+^ T cells, therefore initiating an effective adaptive anticancer immune response. Studies have shown that the therapeutic benefits of Trojan bacteria on GBM are superior to those of essential bacteria. The “Trojan” bacterial system is expected to speed up the development of new treatments for many central nervous system disorders.

Engineering microbes for tumor-targeted treatment has a history of more than a century. In recent years, many synthetic biology techniques have enabled the release of antitumor medicines from designed bacteria at the tumor site, enabling efficient antitumor treatment (Juarez et al., 2022). Utilizing the chemotactic impact of modified bacteria to provide tumor-targeted medication delivery may be one of the most effective methods for addressing the abovementioned issues (Nagata et al., 2022). In the early stage, Ze et al. used the targeting of designed Salmonella and the recruitment of bacteria to neutrophils based on anti-tumor engineering bacteria (Mi et al., 2022). They created a bacterial medication delivery system encapsulating chemotherapeutic medicines (DOX) in bacterial outer membrane vesicles (OMV), enabling tumor-specific drug delivery and boosting glioma treatment. Infused bacteria preferentially colonize hypoxic glioma tumor tissues and carry out three functions: repolarization of M2 macrophages to the M1 phenotype, downregulation of P-gp protein on glioma cells, and recruitment of neutrophils. It can cross the blood-brain barrier of chemotherapy medications, enhancing the tumor immune microenvironment, and increasing the susceptibility of tumors to chemotherapy treatments, thus killing three birds with one stone. This research produced an effective glioma therapy, a novel therapeutic approach for treating glioma. This combination anti-tumor technology platform provides fresh concepts for creating more combined tumor treatment techniques, which is the foundational effort for clinical transformation.

## Conclusion

Glioma is the most prevalent malignant central nervous system tumor. It has an aggressive growth pattern, high invasiveness and proliferative capacity, poor surgical therapeutic impact, and a low patient survival rate (Bota et al., 2022). To restrict the growth of glioma, it will be crucial for future research to identify direct and closely related therapy techniques or to help establish treatments(Martins et al., 2023). With microorganisms proving effective in treating tumor instances, there is reason to be optimistic about the future of cancer therapy. Bacteria-based therapy offers excellent oncolytic potential, can be administered precisely, and has high specificity. It may effectively reverse cancers by expressing a wide range of anti-tumor proteins in both high-quality and large quantities (Chen et al., 2023b; Wang et al., 2023a). In summary, when it comes to pinpointing cancerous cells, bacterial-based therapies far outperform more traditional methods. Following genetic engineering or membrane modification, OMVs released by bacteria may be employed as carriers of anticancer medicines or other immunomodulators (Hu et al., 2022; Suri et al., 2023).

Furthermore, the innovative combination of nanomaterials and bacteria may boost tumor therapy efficiency while considerably reducing adverse effects on the human body. This is because nanoparticles work well as chemotherapeutics and are naturally drawn to bacteria (de Souza-Guerreiro et al., 2023). While the curative promise of genetically altered bacteria is undeniable, there are still obstacles to overcome when employing these bacteria in an actual therapy setting. There are various issues with genetically modified bacteria in clinical applications, including toxicity, DNA instability, unchecked growth, ineffective targeting, off-target infection, and inadequate drug output(Chen et al., 2022b; Zhu et al., 2022). Studies using bacteria-mediated tumor treatment are currently in phase I or phase II clinical testing. With the constant advancement of gene technology, it is predicted that this kind of “smart” bacteria will become a potent weapon for humans in the battle against cancerous tumors (Pan et al., 2021; Wang et al., 2023b). Informed conjecture on our part suggests that adding tumor-specific bacteria to cancer medication may open up new avenues for treating glioma.

## Author contributions

GZ and JS prepared figures and tables. YM designed and wrote the manuscript. YM, TY, and HP contributed to the critical revision of the English language. YM was responsible for its financial support. All authors approved the final manuscript. All authors contributed to the article.
